# Feasibility, Acceptability, and Impact of a Self-guided e-Learning Memory Program for Older Adults

**DOI:** 10.1093/geroni/igab046.3492

**Published:** 2021-12-17

**Authors:** Danielle D'Amico, Iris Yusupov, Lynn Zhu, Jordan Lass, Cindy Plunkett, Brian Levine, Angela Troyer, Susan Vandermorris

**Affiliations:** 1 Ryerson University, Toronto, Ontario, Canada; 2 York University, Toronto, Ontario, Canada; 3 Baycrest, Toronto, Ontario, Canada; 4 Baycrest, North York, Ontario, Canada

## Abstract

Clinician-led memory interventions have been shown to increase knowledge, reduce anxiety, promote memory-strategy use, and increase brain-healthy lifestyle behaviours in older adults with normal age-related memory changes. A self-guided, e-learning version of the Baycrest Memory and Aging Program® was recently developed to increase accessibility to memory interventions. The objectives of the current study were to assess program feasibility (retention rate), acceptability (satisfaction), and participant-reported impact (memory concerns, behaviour change, goal attainment). As part of a larger study, participants were 139 healthy older adults (mean age: 73±7, 73% female). Ninety-two individuals completed the program (retention rate=66%). Anonymous feedback data indicated a high level of satisfaction with the program overall (98%), the pace and clarity of the learning modules (100%), and the organization and navigation of the interface (92%). Suggested improvements included offering more interaction with others and addressing minor platform glitches. There was a decrease in the level of concern about memory change, with 64% expressing concern at a level consistent with the Jessen et al. (2014) criteria for Subjective Cognitive Decline at baseline, and 23% expressing the same at post-test. The majority of participants reported increases in using memory-strategies (63-97%) and lifestyle-promoting behaviours (40-72%). All participants reported moderate to high satisfaction with personal goal attainment. Results support feasibility, acceptability, and impact of a self-guided e-learning adaptation of memory intervention. E-learning tools may be a promising avenue to deliver accessible brain health promotion in later life, especially in the context of the shift to virtual care during and beyond COVID-19.

